# Sampling in epidemiological research: issues, hazards and pitfalls

**DOI:** 10.1192/pb.bp.114.050203

**Published:** 2016-04

**Authors:** Stephen Tyrer, Bob Heyman

**Affiliations:** 1 Newcastle University, UK; 2 University of Huddersfield, UK

## Abstract

Surveys of people's opinions are fraught with difficulties. It is easier to obtain information from those who respond to text messages or to emails than to attempt to obtain a representative sample. Samples of the population that are selected non-randomly in this way are termed convenience samples as they are easy to recruit. This introduces a sampling bias. Such non-probability samples have merit in many situations, but an epidemiological enquiry is of little value unless a random sample is obtained. If a sufficient number of those selected actually complete a survey, the results are likely to be representative of the population. This editorial describes probability and non-probability sampling methods and illustrates the difficulties and suggested solutions in performing accurate epidemiological research.

It is wise to be chary of surveys and polls, and to always read the figures carefully. In the heady excitement just before the vote on Scottish independence many observers thought that allowing teenagers to vote would propel Scotland to self-government. After the referendum, the *Straits Times* in Singapore stated ‘Young people voted in droves to break up the centuries-old union’, based on an exit poll that showed that 71% had voted ‘yes’ to independence.^1^ This poll only included the very small number of 14 people in this age bracket, 4 of whom had voted ‘no’. A later, more representative YouGov poll with a much larger sample reported that 51% of 16- to 24-year-olds had voted ‘no’.^2^ In whatever way the first sample had been selected, its small size would have made it highly susceptible to sampling error. When there is a strong expectation that a particular event is going to result there is a strong inclination to believe the anticipated outcome.

## Sampling in epidemiological studies

Sampling for health-related research does not usually need to be as precise as sampling for political surveys but in epidemiological investigations every effort should be made to select a representative sample. Often this is not achieved. Concern has been expressed for years about the number of prisoners who have mental health problems. In a 1979 study in the USA to estimate the prevalence of mental illness in prisoners, 33 male prisoners were selected and interviewed by a psychiatrist using an instrument called the Psychiatric Status Schedule.^3^ Of those interviewed 3% were diagnosed as having a mental disorder and 27% had a drug or alcohol problem.^4^ The main problem with this paper is the number of people sampled and how they were selected. Although it is stated that the prisoners were selected at random, the number of prisoners selected for interview is on the low side. The procedure for randomisation is not indicated. Female prisoners were not included. The determination of the prevalence of mental illness from a survey in one prison in one state in the USA cannot be extrapolated to the whole country, where there are more than six grades of prisons according to the degree of security required. There is no indication in the paper about how the sampling procedure controlled for the proportion of inmates that were detained and those that were sentenced. Apart from sampling errors, justifiable criticism can also be made of the reliability of only having one psychiatrist reviewing all prisoners, the categorical method of diagnosis (mental disorder or drug or alcohol misuse) and the use of the Psychiatric Status Schedule, which is reported to have consistency in many of its scales. Under these circumstances it is unsurprising that the estimate of prevalence of mental disorder in this survey did not accord with a recent systematic review examining studies over a 40-year period which found 14% of prisoners had a diagnosed psychiatric disorder.^5^

When carrying out any survey of any type it is essential for the researcher to clearly define the target population that they wish to sample. On some occasions the population will be sufficiently small, and the researcher is able to include the entire population in the study. This is termed a census study. Much more frequently the population is too large for all its members to be contacted and so a sample is chosen to reflect the characteristics of the population from which it is drawn.

## Sampling methods

Sampling methods are described as either probability or non-probability methods (Box 1).^6^ In probability samples, each member of the population has an exactly equal chance of being selected. Types of probability sampling include random sampling, stratified and systematic sampling. Probability sampling is a more accurate method in determining the true characteristics of the population but it is not perfect. Sampling error refers to the variations from the true population parameter which can result from random sampling. With true probability samples sampling error is reduced by having larger samples. In non-probability sampling, the degree to which the sample differs from the population is unknown.

**Box 1** Sampling methods

Census study: whole population under enquiry

Probability sampling:

random
systematic
stratified

Non-probability sampling:

convenience
judgment
quota
snowball

Qualitative research: purposive

### Sample size

To estimate how large the sample should be to reflect the total population the confidence level of the mean of the results, a measure of the variance of the responses of the sample (standard deviation) and an estimate of the margin of allowable error need to be determined. The calculation is not difficult and help can be readily accessed (www.qualtrics.com/blog/determining-sample-size).

## Types of probability sampling

### Random sampling

In random sampling every member of the population has the same chance (probability) of being selected into the sample. Using a random sample it is possible to describe quantitatively the relationship between the sample and the underlying population, giving the range of values, called confidence intervals, in which the true population parameter is likely to lie. Random does not mean arbitrary. Choosing a random sample relies on an objective mechanism to select elements from the population. This is usually done by a computer, but rolling dice or using random numbers are also acceptable options.

### Stratified and systematic sampling

Stratified sampling is often used when one or more of the strata (subsets of the population) have a low incidence relative to the other strata. It can also be used to reduce sampling error.

In systematic sampling every 5th, 10th, 20th or *n*-th record is selected from a list of population members. It is no more than a form of random sampling.

## Non-probability sampling

In non-probability sampling members are selected from the population in any form of non-random manner. Examples include convenience sampling, judgement sampling, quota sampling and snowball sampling.

### Convenience sampling

Convenience, accidental or opportunistic sampling is used to find out a cheap estimate of the truth. An easily accessible non-random selection of the population under enquiry is chosen. A frequently used method is contacting people by email.

### Judgement sampling

An extension of convenience sampling is judgement sampling. Thus, when carrying out a national enquiry on the frequency of depressive illness, one specific town and one rural area that are thought to be typical of the country as a whole may be selected. Ideally, the chosen sample needs to be representative of the entire population and this is difficult to determine.

### Quota sampling

Quota sampling is the non-probability equivalent of stratified sampling. In the first instance the investigator identifies the strata and their frequency in the population. Convenience sampling is then used to select the required number of participants from each stratum.

### Snowball sampling

Snowball sampling is a special non-probability method used when there are difficulties in identifying members of the population or if the desired sample characteristic is rare. This technique relies on existing study participants recruiting future participants from among their acquaintances. It is often used when it is anticipated that individuals may be reluctant to be identified, for instance when surveying illegal drug users. Although inexpensive, major bias may result because a balanced cross-section of the population is not identified.

## Which sampling method to use?

Which sampling method to use depends on the nature of the survey proposed. Epidemiological research requires a representative sample but there is a great deal of health research that does not need one. Service evaluations and randomised controlled trials (RCTs) do not require a survey design. In an RCT the main purpose is to compare groups within the sample, members of which are placed into them randomly, such as treatment *v*. placebo. Similarly, health psychometrics (e.g. design of health measures), experimental studies, theoretical-based research studies (e.g. testing a theory or proposing a new theory), observational studies (e.g. looking for relationships of theoretical constructs, such as depression and self-esteem) are mostly conducted using opportunistic samples. Precisely accurate statistics may not be required.

Qualitative researchers are often concerned with what exists rather than how much,^7^ and seek to delve into complex processes such as responding to long-term illnesses. Purposive sampling, one of the most common sampling strategies, groups participants according to pre-selected criteria relevant to a particular research question. There are more: Kuzel^8^ identified 13 different forms of qualitative sampling strategy, including maximum variation, theory-driven, critical case and deviant case. One case is sufficient at times to illustrate a point. For example, Heyman *et al*^9^ explored the experiences of a female patient who had ‘risked exploding’, according to a colorectal nurse, by absconding from hospital to have sexual intercourse with her boyfriend immediately after anal cancer surgery. The aim of the study was to understand why one particular individual had behaved in such a medically risky and highly unusual way. A recent introduction to qualitative research methodology is provided by Silverman.^10^

## Hazards of non-probability sampling

When performing a survey there is a strong temptation to obtain information from as much of the population as possible in the belief that accuracy can be increased in this way. An example is given to show that this may be fallacious.

Many of us are interested in psychiatrists' views about service issues. A researcher wishes to find out the opinions of psychiatrists about policy regarding controlled drugs. A questionnaire is designed with a number of statements ranging from tighter control over existing drugs to decriminalisation of all unscheduled agents. Respondents have to select which statement best accords with their views. The researcher is also interested in the responses of grades of psychiatrist to see whether there are different attitudes about the issue between consultants and trainee psychiatrists. The Royal College of Psychiatrists holds the names of all psychiatrists in the UK, and the researcher is given access to this list. It is proposed that as many psychiatrists as possible are required, and so all the psychiatrists are contacted by email and asked their views. When all the questionnaires are returned online the response rate is 38% with 5128 psychiatrists completing the questionnaire. The analysis of the replies of this large number of people takes a good deal of time but this is completed after a few months and the paper is written. It is submitted to a prestigious psychiatric journal and is rejected. What were the reasons?

A proportion of the individuals would not have been contactable by email, and this group may have different attitudes from the rest. The nature of the responses of those individuals who failed to reply to the questionnaire, the majority, is unknown. They might have differed from respondents if, for instance, busier or more stressed psychiatrists were less likely to participate. As a result, the sample identified by the researcher may not have been representative and the findings cannot be safely generalised to all those working in this field. This is a non-probability sample and, as such, statistical inferences cannot be validly made from the results. Notwithstanding, the results of this survey are not valueless. Although they cannot be reliably generalised to the total population of psychiatrists, they could still be useful for piloting purposes. Certain questions on the survey could be refined and/or alternative questions included in a later enquiry.

## How to conduct a probability sample

In the example referred to above the sample size should be determined (see earlier) and the names of those selected for interview entered into a sampling frame. Attempts should be made to contact all those included to ensure that the results are representative. Multiple efforts must be made to persuade those selected to complete the survey questionnaire. If most of the initially identified sample do provide information, the results can be analysed statistically and valid conclusions can be drawn.

The researcher will need to decide whether to aim for a simple probability sample or to stratify the sample by predetermining the numbers to be selected randomly into relevant categories, for example, in this case, occupational grade (consultant, specialist registrar, etc.), gender. Stratification ensures that the sample is representative of the population with respect to the chosen population parameters if known; or, more commonly, to ensure that categories with smaller numbers in the population (e.g. associate specialists) are adequately represented for comparative purposes. An introduction to stratified and other forms of complex probability samples is provided by Bryman.^11^

### Selection bias

Selection bias can arise if insufficient numbers of individuals identified in the sampling frame fail to complete the questionnaire. The greater the number of non-respondents who fail to complete the exercise the more scope there is for the sample to be skewed in an unknown direction. As a rule of thumb, the researcher should aim for at least a minimum of 60% completion by those selected from the sampling frame and every effort should be made to achieve more than this. If the percentage of those completing the questionnaire is less than 100%, as it almost invariably will be, there are a number of strategies the investigator can adopt to manage non-response bias.

### Avoiding non-response bias

In the first instance, the non-respondents should be approached asking them again to complete the questionnaire. In those who fail to respond again a third attempt should be made to urge them to reply. Comparisons can then be made between first-, second- and third-time responders. If the responses are similar then extra sampling may not be needed. If the responses of the late respondents are very different to the rest of the study then it may be necessary to contact more of the non-respondents. This depends on the proportion of respondents completing the survey, the larger the number the better.

It may not be necessary to obtain more data as it has been shown that the observations of late responders are more like non-responders than are first-time responders,^12^ so the responses of the late responders can be applied to those who failed to respond to the enquiry. This cannot be assumed, however, and late respondents in some surveys behave like earlier participants.^13^

It has also been shown that if a small random sample of non-respondents is selected and all can be contactable and complete the survey, the results can be extrapolated to the remainder of the non-respondents. The relatively small number of 20 is considered to be sufficient for this purpose if all complete the questionnaire.^14^ In practice, it is very difficult to ensure such a 100% response in a survey of this nature and this aim may not be achievable.

We hope this article will persuade the reader to examine the methods that have been used to perform surveys of opinions and other issues. Let us quote a final example. A *Mail On Sunday* poll in August 2011 showed that the majority of those surveyed backed the reintroduction of capital punishment.^15^ One thousand people took part in this survey which was said to be representative of British public opinion. The consumer panel from which these people were selected were contacted online so those without email access were not included. Furthermore, members of this panel are paid for a registration of their interest and for each poll in which they give their opinion. They are possibly representative of the *Daily Mail* readership but not of the general population whose views may or may not correspond to those of the sample.

Those intending to perform surveys can find more information in this document: www.sagepub.com/upm-data/40803_5.pdf. Those wishing to carry out surveys on psychiatric topics, particularly if involving the membership of the Royal College of Psychiatrists, should contact the College Registrar.
